# Predicting insulin use among women with gestational diabetes diagnosed in oral glucose tolerance test

**DOI:** 10.1186/s12884-023-05746-8

**Published:** 2023-06-02

**Authors:** Tatiana A Zaccara, Fernanda C F Mikami, Cristiane F Paganoti, Rossana P V Francisco, Rafaela A Costa

**Affiliations:** 1 Departamento de Obstetrícia e Ginecologia da Faculdade de Medicina da Universidade de São Paulo, Sao Paulo, SP Brazil; 2 Divisão de Clínica Obstétrica do Hospital das Clínicas da Faculdade de Medicina da Universidade de São Paulo, Sao Paulo, SP Brazil

**Keywords:** Gestational diabetes, Oral glucose tolerance test, IADPSG, Insulin, Diet

## Abstract

**Background:**

Gestational diabetes mellitus (GDM) is one of the most common complications affecting pregnant women. While most women will achieve adequate glycemic levels with diet and exercise, some will require pharmacological treatment to reach and maintain glucose levels between the desired thresholds. Identifying these patients early in pregnancy could help direct resources and interventions.

**Methods:**

This retrospective cohort of women with GDM diagnosed with an abnormal 75g-OGTT presents data from 869 patients (724 in the diet group and 145 in the insulin group). Univariate logistic regression was used to compare the groups, and multivariable logistic regression was used to identify independent factors associated with the need for insulin. A log-linear function was used to estimate the probability of requiring pharmacological treatment.

**Results:**

Women in the insulin group had higher pre-pregnancy BMI index (29.8 vs 27.8 kg/m^2^, odds ratio [OR] 1.06, 95% confidence interval [CI] 1.03–1.09), more frequent history of previous GDM (19.4% vs. 7.8%, OR 2.84, 95% CI 1.59–5.05), were more likely to have chronic hypertension (31.7% vs. 23.2%, OR 1.54, 95% CI 1.04–2.27), and had higher glucose levels at all three OGTT points. Multivariable logistic regression final model included age, BMI, previous GDM status, and the three OGTT values as predictors of insulin requirement.

**Conclusions:**

We can use regularly collected data from patients (age, BMI, previous GDM status, and the three OGTT values) to calculate the risk of a woman with GDM diagnosed in OGTT needing insulin. Identifying patients with a greater risk of requiring pharmacological treatment could help healthcare services to better allocate resources and offer closer follow-up to high-risk patients.

**Supplementary Information:**

The online version contains supplementary material available at 10.1186/s12884-023-05746-8.

## Background

Gestational diabetes is the most common complication affecting pregnant women, with prevalence varying around the world [1]. According to the International Diabetes Federation, 15.8% of the live births in 2019 were affected by hyperglycemia, and 83.6% of these births were due to gestational diabetes mellitus (GDM) [2].

Following the Hyperglycemia and Adverse Pregnancy Outcomes (HAPO) study in 2008 [3], the International Association of Diabetes and Pregnancy Study Groups (IADPSG) in 2010 proposed a set of criteria in an attempt to make diagnostic uniform worldwide [4]. However, adopting these criteria led to an increase in GDM prevalence [5, 6] and impacted the health services which adopted them since patients with GDM usually require specialized care and more frequent prenatal visits as well as closer monitoring during peripartum and postnatal periods.

The combination of diet and exercise will suffice to achieve adequate glycemic control for 70–85% of the women diagnosed with GDM, but some will need pharmacological interventions to maintain glucose levels below the recommended thresholds [7]. Identifying women at greater risk of needing insulin could help caregivers to provide intensive education and counseling to patients diagnosed with GDM and help healthcare centers to distribute resources accordingly to their specific needs.

This study aimed to use routinely collected data to identify risk factors associated with insulin need in women with gestational diabetes and to develop an easy-to-use application to estimate the probability of insulin need.

## Methods

**Fig. 1:**
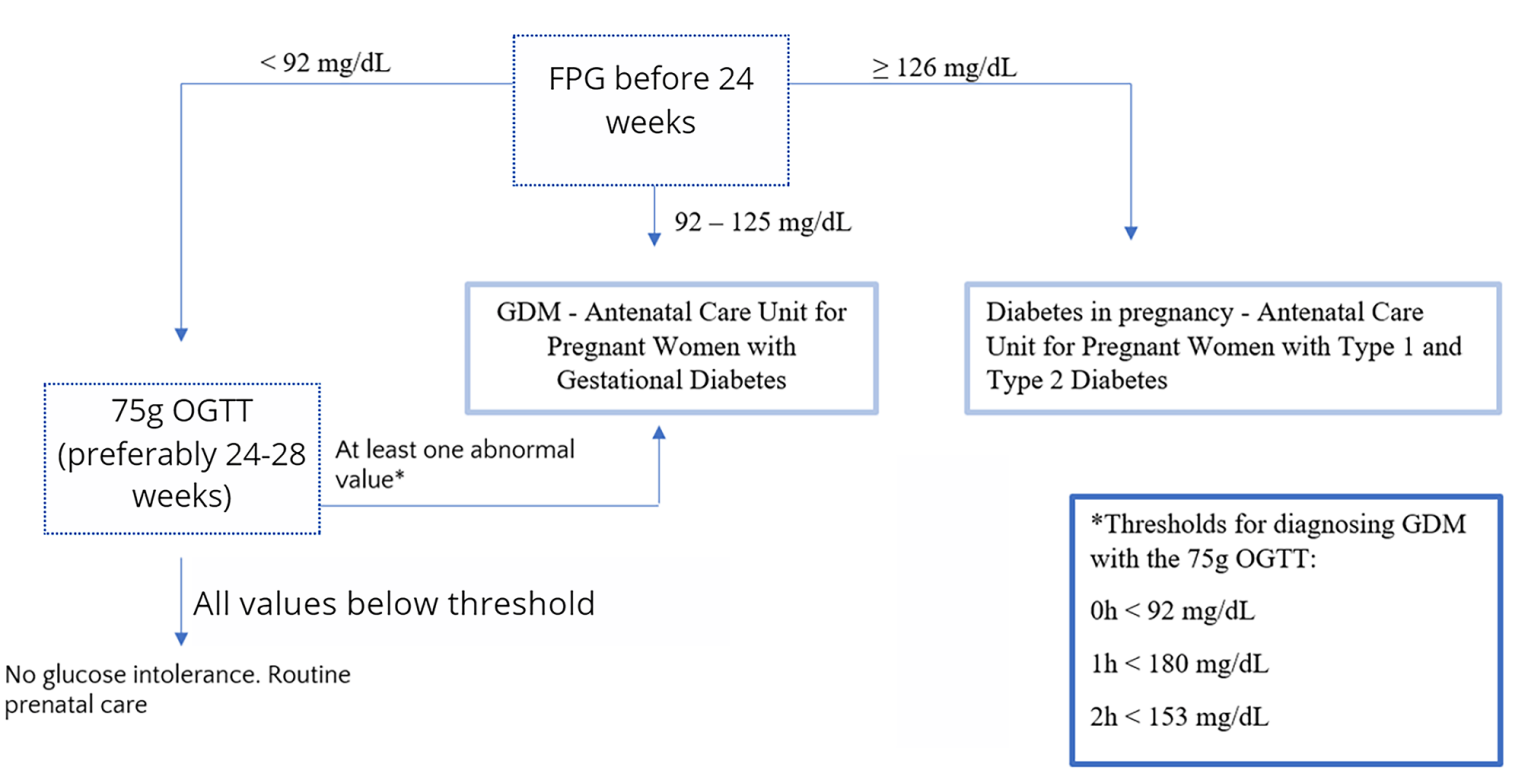
Diagnosis of Gestational Diabetes in the Obstetrics Department of Hospital das Clinicas – Universidade de Sao Paulo

This is a cross-sectional study nested in a cohort of women followed up in the Gestational Diabetes Unit in the Obstetrics Department of Hospital das Clinicas da Faculdade de Medicina da Universidade de Sao Paulo (Sao Paulo – Brazil). Medical records of all patients with a singleton pregnancy who attended prenatal care in our hospital between 01/01/2012 and 03/31/2020 with abnormal glucose levels on 75g-OGTT (75 grams oral glucose tolerance test) performed between 24 and 32 weeks of gestational age were reviewed. The criteria for diagnosing gestational diabetes in our department are shown in Fig. 1. We compared patients who needed insulin to achieve adequate glycemic control and those who maintained satisfactory glycemic levels with diet and exercise only. Patients with a diagnosis of type 1 or type 2 diabetes were not included in this study.

An antenatal specialized multidisciplinary team of doctors, nurses, and dietitians follows all patients diagnosed with GDM. They receive lifestyle changes and nutritional recommendations and are instructed to perform self-monitoring blood glucose measurements at least four times per day. Glucose targets in our service are the ones recommended by American Diabetes Association: fasting glucose value < 95mg/dL, 1-hour postprandial glucose value < 140mg/dL, and 2-hour postprandial < 120 mg/dL. If diet and exercises are not enough to achieve adequate glycemic control, insulin is the first option for pharmacological treatment.

Electronic charts were reviewed, and we obtained the following data for each participant: age, pre-pregnancy body mass index (BMI), parity, history of GDM, family history of DM, connective tissue disease, chronic hypertension, asthma, smoking habit, FPG at the first appointment, gestational age at the screening 75g-OGTT, glucose levels on the OGTT, and use of insulin during pregnancy.

The study was conducted after approval by the Ethics Committee of Hospital das Clinicas – FMUSP, Sao Paulo, Brazil (approval number: 48868915.9.0000.0068). Informed consent was waived because of its retrospective nature. All methods were carried out under relevant guidelines and regulations.

### Statistical analysis

Patients were divided into two groups according to the treatment needed to maintain adequate glycemic control: diet and insulin. Clinical and laboratory data between the groups were compared and are presented as mean and standard deviation, or absolute numbers and percentages, as appropriate.

Univariate logistic regression was used to estimate odds ratios (OR) and their respective 95% confidence intervals (CI) for comparison between the diet and insulin groups. A multivariable logistic regression analysis was performed following this analysis, including statistically significant variables from the univariate logistic regression and clinically relevant variables, to identify independent factors associated with the need for insulin. A log-linear function was used to estimate the probability of needing insulin.

Statistical software SPSS version 26 (IBM SPSS Inc., Armonk, NY, USA) was used for statistical analysis, and a *P*-value < 0.05 was considered statistically significant.

## Results

Between January 1, 2012, and March 31, 2020, 2235 patients were followed-up at the gestational diabetes unit. Of these, 1115 had abnormal FPG levels early in pregnancy, and 1120 had abnormal OGTT results. We excluded 39 patients with multiple pregnancies, 188 patients with the diagnostic test done before or after the period of 24–32 weeks, six patients with no recorded date of the diagnostic test, and 18 patients whose records did not have information about treatment. The remaining 869 patients were included in this analysis. They were classified into two groups according to the treatment needed to achieve adequate glycemic control: 724 in the diet group (83.3%) and 145 in the insulin group (16.7%).

The baseline characteristics of each group are presented in Table 1. Women in the insulin group had higher pre-pregnancy BMI values, a more frequent history of GDM in a previous pregnancy, and were more likely to have chronic hypertension. The glycemic values in these women’s OGTT were also higher in all three measurements (fasting, 1 hour after glucose intake, and 2 hours after glucose intake). There were no statistically significant differences regarding family history of diabetes, connective tissue diseases, asthma, or smoking habit.

**Table 1:** Baseline characteristics of women followed-up in the Gestational Diabetes Unit, classified according to their treatment

Variable (n of recorded data)	Diet (n = 724) Mean ± SD N (%)	Insulin (n = 145) Mean ± SD N (%)	*Odds ratio* (95% CI)
Age, years (n = 869)	32.6 ± 6.3	33.5 ± 5.8	1.03 (1.00–1.06)
BMI, kg/m^2^ (n = 854)	27.8 ± 5.8	29.8 ± 6.4	1.06 (1.03–1.09)
Primigravida (n = 869)	225/724 (31.1%)	37/145 (25.5%)	0.76 (0.51–1.14)
Family history of DM (n = 869)	382/724 (52.8%)	80/145 (55.2%)	1.10 (0.77–1.58)
Previous GDM (n = 605) (for non-primigravida patients)	39/497 (7.8%)	21/108 (19.4%)	2.84 (1.59–5.05)
Connective tissue disease (n = 851)	31/708 (4.4%)	6/143 (4.2%)	0.96 (0.39–2.34)
Chronic hypertension (n = 869)	168/724 (23.2%)	46/145 (31.7%)	1.54 (1.04–2.27)
Asthma (n = 716)	29/597 (4.9%)	7/119 (5.9%)	1.22 (0.52–2.86)
Smoking habit (n = 864)	42/719 (5.8%)	8/145 (5.5%)	0.94 (0.43–2.05)
Fasting glucose at the first appointment (mg/dL) (n = 763)	80.8 ± 7.0	82.2 ± 7.1	1.03 (1.00–1.06)
Gestational age–OGTT (weeks + days) (n = 869)	27w0d ± 14d	26w4d ± 13d	0.98 (0.97–1.00)
Fasting plasma glucose-OGTT (mg/dL) (n = 869)	89.3 ± 10.5	98.1 ± 13.6	1.07 (1.05–1.09)
1-h plasma glucose (1h-PG) (mg/dL) (n = 852)	160.9 ± 30.7	180.4 ± 37.2	1.02 (1.01–1.03)
2-h plasma glucose (2h-PG) (mg/dL) (n = 854)	150.7 ± 29.0	167.7 ± 39.6	1.02 (1.01–1.02)

SD, standard deviation; CI, confidence interval; BMI, body mass index; DM, diabetes mellitus; GDM, gestational diabetes mellitus; OGTT, oral glucose tolerance test

When analyzing independent risk factors for insulin use, we identified that previous GDM represented an almost 3-fold increase in the chance of needing insulin, but this does not apply to primigravida women. Because of this, we used three categories for the variable “Previous GDM” when performing the multivariable logistic regression: “primigravida” (which was the reference category), "no," and "yes."

Multivariable logistic regression analysis was performed, and the final model included age, BMI, previous GDM status, and the three OGTT values. The coefficients of each variable are shown in Table 2.

**Table 2:** Predictors of insulin need for women diagnosed with GDM through the 75g-OGTT taken between 24 and 32 weeks

Parameter	Regression Coefficient	OR (95% CI)	p
Intercept	-11.971		
GDM = primigravida	0	ref	
GDM = no	0.136	1.17 (0.71–1.92)	0.593
GDM = yes	0.983	2.81 (1.37–5.78)	0.007
Age (years)	0.017	10.2 (0.98–1.06)	0.362
BMI (kg/m^2^)	0.037	1.05 (1.01–1.08)	0.025
FPG – OGTT (mg/dL)	0.056	1.06 (1.04–1.08)	0.000
1h-PG (mg/dL)	0.009	1.01 (1.00–1.02)	0.019
2h-PG (mg/dL)	0.011	1.01 (1.00–1.02)	0.004

The probability of requiring insulin during pregnancy to achieve adequate glycemic levels was calculated using the equation shown in Additional File 1.

**Fig. 2:**
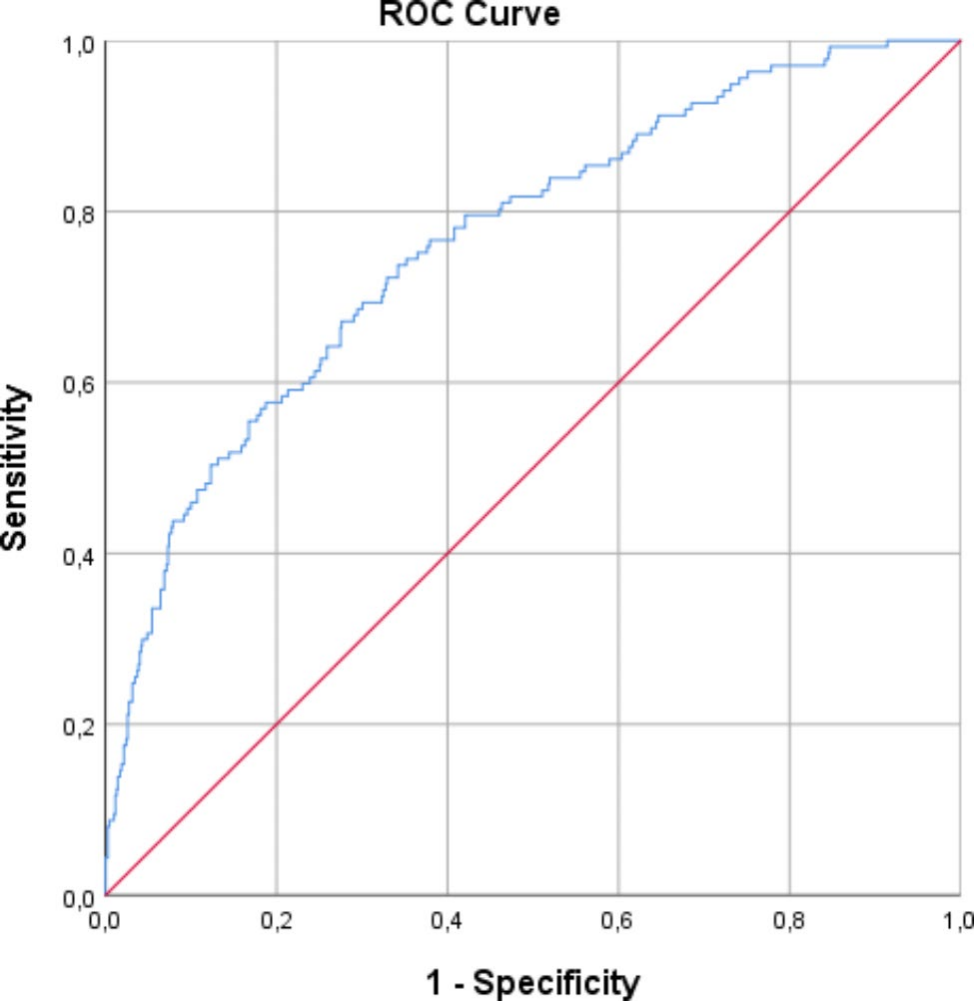
Receiver operating characteristic (ROC) curve for the final multivariable logistic regression model

The final model yielded the Receiver operating characteristic (ROC) curve shown in Fig. 2, with an area under the curve (AUC) of 0.77 (95% CI 0.72–0.81).

The sensitivity vs. specificity graphic is available in Additional File 2; the balance point occurred with a probability of 14%.

Considering pre-specified cut-off points, we calculated our model’s sensitivity, specificity, positive predictive value, negative predictive value, and accuracy. The results are detailed in Table 3.

**Table 3:** Sensitivity, specificity, positive predictive value, negative predictive value, and accuracy for different cut-off points of the model

Cut-off point	Sensitivity	Specificity	PPV	NPV	Accuracy
10%	84,6	50,7	25,2	94,4	56,3
15%	61,8	73,5	31,3	90,7	71,6
20%	50,0	85,4	40,2	89,7	79,6
25%	43,4	90,1	46,1	89,0	82,4
30%	35,3	93,2	50,5	88,0	83,7

## Discussion

Since the adoption of the diagnostic criteria for GDM proposed by IADPSG, many services have been impacted by the increase in gestational diabetes prevalence [5, 6]. Gestational diabetes comprises different levels of hyperglycemia, and most women will achieve adequate glycemic levels with diet and exercise only and could be monitored by primary care providers. On the other hand, patients who use insulin require more prenatal visits, spend more on glycemic monitoring supplies, and demand training and education to safely manage the medication [1, 7].

Previous studies from our service analyzed the data on women with gestational diabetes to predict the risk of needing insulin, but the populations in these studies differ from the one we studied here. Sapienza et al. [8] analyzed women diagnosed with GDM according to the diagnostic approach (100g-OGTT) used before adopting IADPSG criteria. They found that pre-pregnancy BMI, family history of diabetes, number of abnormal values on the test, and glycated hemoglobin levels could be used to estimate the risk of a woman needing pharmacological treatment during pregnancy. Souza et al. [9] analyzed women with abnormal fasting plasma glucose before 24 weeks of gestational age. They found that maternal age, pre-pregnancy BMI, FPG value, prior GDM, and family history of diabetes were predictors of insulin need.

In our cohort, the insulin use rate was 16.7%. This is lower than what is described by Ducarme et al. [10], Tang et al. [11], Du et al. [12], and Ford et al. [13] and higher than what Nishikawa et al. [14] described. It is important to highlight that the screening and diagnostic strategies were not the same in all these studies, which could explain the difference in pharmacological treatment rates.

Women who needed insulin had higher pre-pregnancy BMI, higher values in all three points of the OGTT, a more frequent history of previous GDM, and were more likely to have chronic hypertension. Nevertheless, chronic hypertension did not maintain statistical significance when performing the multivariable logistic regression. The final model included BMI, previous GDM status, and the glycemic values in OGTT. There was no difference between the diet and insulin groups regarding age, connective tissue disease, asthma, and smoking habit.

The findings of higher BMI and a more frequent history of GDM in a previous pregnancy in the insulin group agree with earlier reports that weight and history of GDM are related to the need for pharmacological treatment in women with gestational diabetes [11–14]. Our analysis also concurs with other studies’ findings that glycemic levels in the OGTT were higher among women who needed insulin to achieve adequate glycemic control [10, 11, 13–16].

Some authors found glycated hemoglobin to be related to the need for pharmacological treatment [10–12, 14, 16], but this test is not routinely offered in our department. Glycated hemoglobin is an indirect measure of glycemic levels and can be impacted by conditions that alter the hemoglobin life span, such as pregnancy [17]. Because we wanted to predict insulin use with readily available information and without the necessity to order any additional laboratory tests, we chose not to include glycated hemoglobin in the study.

All variables analyzed in this research are commonly collected during prenatal care, meaning there is no cost increase to implement this analysis. It can be done during routine appointments without any delay for the patient’s treatment, with the advantage that it can direct efforts by the healthcare team to emphasize some aspects of the orientation, like the impact of lifestyle modifications.

To make the use of the probability calculator easier, we built a tool where the user can input the patient’s parameters and calculate the predicted likelihood of that person needing insulin during pregnancy.

The optimal cut-off point depends on the resources of each local. For healthcare settings with very limited availability, a cut-off point of 25% to consider a patient as “high risk” may be adequate. On the other hand, for places with moderate availability, a cut-off point of 15% may be adequate to identify patients with a low risk of needing insulin, making it possible to refer these women to primary or secondary medical care. It can also be used to determine which patients could be referred to group orientations with dietitians and which should receive individual care and close follow-up appointments to improve adherence to lifestyle modifications and possibly reduce the need for pharmacological treatment.

Our study’s strengths are the large number of cases reviewed and the fact that they were treated by the same team, following the same protocol. Some limitations need to be recognized: this was a single-center retrospective cohort, and additional prospective multicentric large studies should be conducted to validate and refine the calculator.

Predicting insulin need could help stratify the hyperglycemia severity for patients with gestational diabetes and could be a starting point for healthcare settings to assign resources towards a more rational allocation in situations of limited capacity.

## Electronic Supplementary Material

Below is the link to the electronic supplementary material

Supplementary Material 1

Supplementary Material 2

## Data Availability

The datasets generated during and/or analyzed during the current study are available from the corresponding author on reasonable request.
