# Supplementary material for: Predicting insulin use among women with gestational diabetes diagnosed in oral glucose tolerance test
Source: BMC Pregnancy Childbirth. 2023 Jun 2;23:410. doi: 10.1186/s12884-023-05746-8 (PMC10236572; doi:10.1186/s12884-023-05746-8)
Supplement: Supplementary file 1 — Supplementary Material 1 [file 12884_2023_5746_MOESM1_ESM.pdf]

Math Equation to calculate the probability of requiring insulin during pregnancy.

$$p = \frac{\mathrm{Exp}[-11.971 + (0, if\ primigravida; 0.136\ if\ no\ previous\ GDM; 0.983, if\ previous\ GDM) + (0.017 * age) + (0.037 * BMI) + (0.056 * FPG)\ + (0.009 * \mathrm{1hPG}) + (0.011 * \mathrm{2hPG})]}{1 +\ \mathrm{Exp}[-11.971 + (0, if\ primigravida; 0.136\ if\ no\ previous\ GDM; 0.983, if\ previous\ GDM) + (0.017 * age) + (0.037 * BMI) + (0.056 * FPG)\ + (0.009 * \mathrm{1hPG}) + (0.011 * \mathrm{2hPG})]}$$
