# Supplementary material for: Predicting insulin use among women with gestational diabetes diagnosed in oral glucose tolerance test
Source: BMC Pregnancy Childbirth. 2023 Jun 2;23:410. doi: 10.1186/s12884-023-05746-8 (PMC10236572; doi:10.1186/s12884-023-05746-8)

Graphic representation of sensitivity and specificity for the multivariable logistic regression model for predicting the need for insulin.

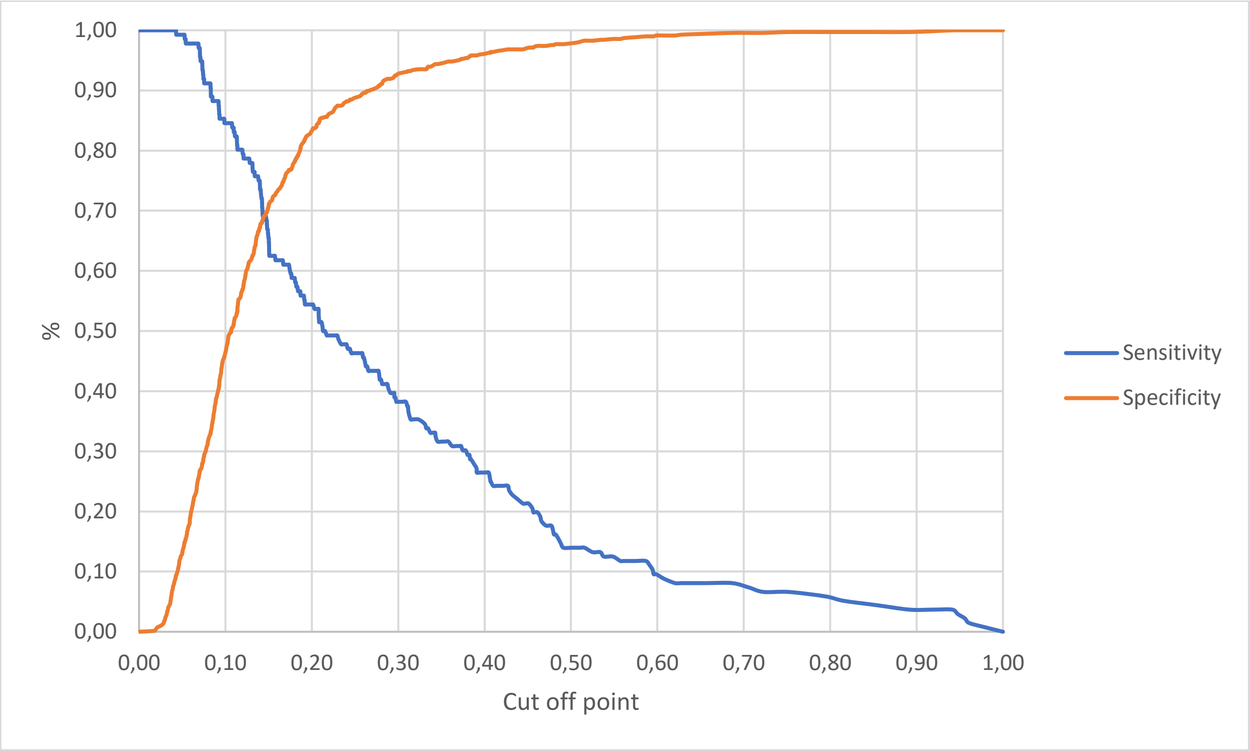

Supplement: Supplementary file 2 — Supplementary Material 2 [file 12884_2023_5746_MOESM2_ESM.pdf]
